# Formation Mechanism of a Coastal Zone Environment Collaborative Governance Relationship: A Qualitative Comparative Analysis Based on fsQCA

**DOI:** 10.3390/ijerph191711081

**Published:** 2022-09-04

**Authors:** Wanjuan Wang, Hongbo Gong

**Affiliations:** Law School, Ningbo University, Ningbo 315211, China

**Keywords:** collaborative governance relationship, coastal zone environment, combination configuration, fsQCA

## Abstract

The coastal zone is an area where terrestrial and marine ecosystems intersect. This region may be subject to outstanding environmental issues, as influenced by many stakeholders. Based on the framework of collaborative governance, the starting conditions for forming a coastal zone environment collaborative governance relationship are proposed as follows: coastal zone environment, balanced level of power and resources, superior-level government participation, and previous cooperation experience. The coastal environmental governance practices of 14 cities along the continental coastal zone of the East China Sea are selected as cases, in order to test the interactions between and influence mechanisms of the starting conditions. As qualitative comparative analysis (QCA), based on set theory and Boolean algebra, is a popular tool to explain complex collaboration situations in small-N cases; and as fuzzy-set qualitative comparative analysis (fsQCA) allows for fine classification of the membership degree (where the condition can be allocated any number between 0 and 1), we use fsQCA to analyze the collaborative governance relationships. The results of the analysis demonstrate that three combination configurations promote the formation of medium–high intensity collaborative governance relationships: high balance level of power and resources × high previous cooperation experience, high pollution of coastal zone environment × high balance level of power and resources × low superior-level government participation, and high pollution of coastal zone environment × high superior-level government participation × high previous cooperation experience. Based on this conclusion, we determine three types of relationship formation modes: wheel-, echo state network-, and umbrella-shaped modes. Notably, under certain conditions, superior-level government participation is not necessary for the formation of a medium–high intensity collaborative governance relationship.

## 1. Introduction

Collaborative governance is increasingly being used to solve global trans-regional environmental problems [1,2]. Effective collaborative governance has been achieved for interstate pollution control in the United States and provincial pollution control in Canada [3]. The coastal zone is the expansion area of the coastline to both sides of the land and the sea, and comprises an important intersection of land and sea ecosystems [4,5]. The coastal zone is an important part of the Earth’s resource system, and it is also an area where ecological environment problems may be concentrated and prominent [6,7,8]. On one hand, the flow of seawater can transport pollutants over long distances [9], the pollutants cannot be limited by administrative demarcation, many government departments and stakeholders are involved [10], and it is necessary to break through administrative divisions and carry out collaborative governance [11,12]. On the other hand, local government departments, enterprises, fishermen, and social organizations may have complex and conflicting interests [13]. For example, the government departments need to develop the economy and protect the environment; enterprises and fishermen need clean coastal environmental resources but may discharge environmental pollutants as well; and environmental protection social organizations need to emphasize their environmental protection objectives and also wish to obtain financial support from the government or enterprises, and their behaviors will have a lasting impact on the coastal environment [14]. Therefore, multi-level cooperation of stakeholders has become a key aspect of coastal environmental governance [15,16]. In practice, faced with the problem of environmental pollution, many countries have established joint pollution prevention and control mechanisms [17,18,19]. However, not all stakeholders can successfully establish medium–high intensity collaborative governance relationships [20,21]. Why can some stakeholders cooperate successfully, while some others cannot? What factors lead to the formation of medium–high intensity collaborative governance relationships?

Collaborative governance involves public–private partnership [22], network governance [23], and inter-local cooperation [24]. These collaborative governance actions are usually initiated as a tool for common goals, which cannot be achieved by any organization alone [25]. The collaborative actions have a brand-new function formed through cooperation [26]; the organic combination of these actors allow the resources and advantages of a single actor to be utilized more effectively [27], and can also reduce costs, disperse risks, and achieve scale benefits through the sharing of human resources, equipment, capital, knowledge, skills, relationships, and so on [28]. In this way, the stakeholders of the coastal environment can establish cross-sectoral cooperative connections for common goals, achieving an overall amplification effect of governance performance achieving “1 + 1 > 2” [29]. Previous studies have focused on the characteristics of stakeholders and the roles that they should play, and some studies have explored the process or design of frameworks for collaborative governance, based on the implicit pre-supposition that collaborative action will naturally occur [21,30]. However, collaboration among stakeholders does not exist naturally and spontaneously, but depends on certain starting conditions [31] or driving factors [32]. These initial conditions greatly impact the success of collaborative environmental governance [33].

Early studies explored the influencing factors of collaborative governance, including past collaborative experience, member dependence and trust, information asymmetry, resource scarcity, failure of a single action, need for risk-sharing, motivation, leadership, and external environmental uncertainty [34,35]. The socio-ecosystem framework (SES) proposes that many variables associated to the resource and governance systems affect the sustainability of cooperative governance of public pond resources [36]. The institutional collective action framework (ICA) states that the authority chooses whether to participate in cooperative governance by measuring transaction costs and collaboration risks [37]. The ecological game theory framework (EGT) has been used to point out that an ecological game based on the cost–benefit considerations of multiple players is key in influencing the establishment of a synergistic relationship [38]. Existing studies have shown that many factors are interweaved and play roles in the formation of collaborative governance relationships [39]. Most existing research has focused on the dispersion of single or multiple influencing factors in collaborative governance [40,41]; however, the combined configuration of the prerequisites for establishing collaborative relationships has not yet been explored sufficiently. When considering coastal zone environments, the governance actors, governance area, and governance scale become increasingly diversified, and the formal identification of single influencing factors can no longer meet the needs of coastal zone environmental governance research. The complex reality of non-linear dynamic governance also requires investigating the interactions of multiple factors. Then, what combination of factors can be used to facilitate collaborative governance of coastal zone environments? With this article, we aim to answer this question, through determining the starting conditions for the establishment of cooperative governance relationships, the results of which can be used as a reference for other coastal areas with similar needs.

The remainder of this paper is structured as follows: The following section presents the theoretical framework and the starting conditions for forming the collaborative governance relationship of the coastal zone environment based on the classical literature. We then screen 14 typical coastal environmental governance practice cases in the continental coastal zone of the East China Sea, obtain case data through formal channels, and calibrate the condition variable data qualitatively through a four-value scheme (i.e., the data are assigned to a fuzzy set with the values: low pollution (0), medium pollution (0.33), high pollution (0.66), and heavy pollution (1)) [42,43]. As qualitative comparative analysis (QCA) is a novel analytical tool that assesses the necessity and sufficiency of conditions in relation to an outcome, it has been used by scholars in recent years to explain complex collaborative governance situations [44,45]. Its fuzzy-set version (fsQCA), based on set theory and Boolean algebra, draws on fuzzy logic [46] and relies mainly on qualitative data sources, offering the possibility to compare intermediate numbers of cases [47]. Therefore, the starting conditions for establishing the collaborative governance relationship of the coastal environment are tested using fsQCA. After that, we explore factor sets that have a significantly positive correlation with the formation of medium–high intensity collaborative governance relationships, analyze the key influencing factors and their combined utility, and construct three modes for the formation. The results of the fsQCA are then summarized and discussed. Finally, the article concludes a discussion of the theoretical and practical implications, as well as the limitations of the study. The key contribution of this paper is identifying different configurations to promote the establishment of multi-agent collaborative relationships in different situations, thus providing a reference for the establishment of different types of collaborative governance regarding coastal zone environments.

## 2. Theoretical Framework

**Figure 1:**
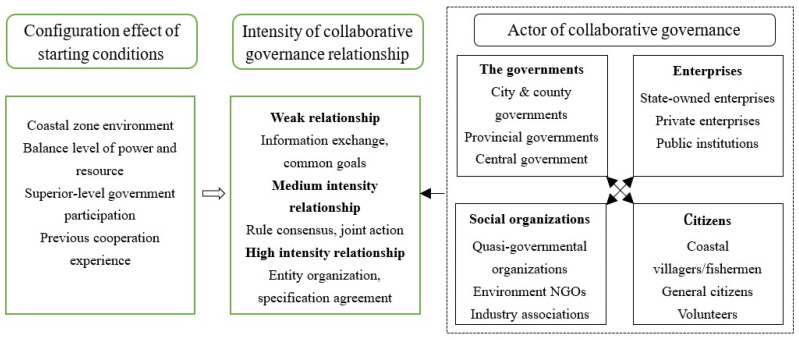
Theoretical framework. Note: “→” represents the connections between various actors of coastal environmental governance and the dashed box denotes the actors in collaborative governance relationships. “⇨” represents the process from condition variables to the result variables. The green rounded rectangles represent the stages of the theoretical framework of this paper, and the green rectangles represent the specific content of the green rounded rectangles.

The theoretical framework of this paper consists of two parts (Figure 1). The first part defines the concept of “relationship formation of collaborative governance of coastal zone environment”, which is constructed by the governments, enterprises, social organizations, and citizens (the connections of actors are indicated by the dashed box on the right side of the figure), and establishes the relationship intensity measurement method (the green box in the middle of the figure). 

The second part proposes four factors that influence the establishment of collaborative governance relationships based on synergy theory, including the coastal zone environment, a balanced level of power and resource, superior-level government participation, and previous cooperation experience (the green box on the left of the figure). It explains how each factor influences the establishment of medium–high intensity collaborative governance relationships.

### 2.1. Formation of the Collaborative Governance Relationship of a Coastal Zone Environment

Collaborative governance of a coastal zone environment is a collective action involving multiple actors, including governments, enterprises, social organizations, and citizens [48,49,50]. The intensity of relationships among multiple actors varies. There are informal relationships, connected only through informal social networks such as a telephone and Internet communication, informal forums, and meetings; as well as formal relationships that engage in substantive interactions, such as cross-organizational networks and public or private meetings through the establishment of specialized cooperation agencies [51]. In informal relationships, simple information sharing and exchanges occur, and a relatively grand or abstract common goal is initially formed [52]; however, there is no specific coordinated action. Formal relationships are often accompanied by the iterative process of “discovery, definition, review, and determination” of governance issues, forming a program of joint action, clarifying the mode and scale of collaborative activities, and resolving conflicts and creating value from work within the jurisdiction of each actor [53], which is the premise of effective collaborative governance. The criterion for forming a formal collaborative governance relationship is whether the joint conference, leading groups, public forums, or collaboration groups have been established and are dedicated to solving problems. A cooperative organization and standardized cooperation agreements must be established [54]. Based on the above, we distinguish three grades of coastal zone environmental collaborative governance relationships (Table 1): weak collaborative relationships, medium intensity collaborative relationships, and high intensity collaborative relationships.

### 2.2. Factors Influencing the Formation of Collaborative Governance Relationship

Ansell and Gash have proposed a theoretical framework for the starting conditions of coordination, believing that the balance level of power and resource, incentives to participate, and history of antagonism or cooperation are critical starting conditions [31]. These three categories are relatively broad and include the conditions involved in other frameworks, such as social capital and mutual trusts. Besides these, some important influencing factors have also been found in water environment and marine research in recent years, including the previous interactions and relationships of participants [57], power imbalances [58], the centralized leadership of the government [59], and resource sharing [60]. Therefore, combined with the influencing factors found in recent empirical research, we use the theoretical framework of Ansell and Gash for reference, and modify it based on the practice of coastal zone environmental governance in China. 

First of all, in China’s authoritarian administrative system, stakeholder collaboration is usually carried out with the approval or support of the superior-level government [61]. Therefore, adding “superior-level government participation” as a leadership variable is necessary. Secondly, China has a vast territory, with a coastline of more than 18,000 km on the mainland and 14,000 km on islands, spanning 10 coastal provinces from the north to the south [62]. The coastal zone environmental conditions in different regions significantly differ [63,64]; moreover, the stronger the spillover of pollutants, the more significant the trans-regional governance effect [65], such that there will also be significant differences in environmental governance between different regions. Therefore, the objective variable “coastal zone environmental conditions” was added. At the same time, as the QCA method can only deal with section data having relatively stable condition variable values, and the variable “participation motivation” mentioned by Ansell and Gash is highly subjective and dynamic, it is difficult to assign a stable value to this variable. Furthermore, the variable “participation motivation” is less significant for the coastal zone environment, the balance of power and resource, the superior-level government participation, and previous cooperation experience. Therefore, it was not considered suitable to be included in the condition variable framework together with the other variables. Therefore, we propose four key starting conditions for forming coastal zone environment collaborative governance relationships: the coastal zone environment, the balance of power and resources, superior-level government participation, and previous cooperation experience. 

#### 2.2.1. Coastal Zone Environment

The structural tension of the environment is the main factor influencing the collective action of environmental governance [66]. The pressure mechanism brought by the discharge of highly polluted wastewater will promote more active collaborative governance with regard to the water environment [67], and stakeholder perception of environmental pollution promotes their motivation to participate in environmental governance [68]. Coastal cities have different pillar industries and economic and social development levels. The types and levels of emissions of pollutants also differ. In addition, the diffusion of environmental pollution in coastal zones follows its own natural laws, where the movement of ocean currents and organisms also affect the dilution, transport, and transformation of pollutants, making the spatial and temporal distribution of pollutants significantly differ between each region, resulting in varying environmental governance interests in each region.

#### 2.2.2. Balance Level of Power and Resources

“Coordination” means that actors need to be open to each other. The difference in power and resource level is often accompanied by a difference in governance capacity. When multiple actors are in an unbalanced power and resource system, the strong side is more likely to occupy an active position, coercing the weak side to achieve its own goals [69], thus affecting the willingness and effectiveness of the weak side to participate in governance. Stakeholders are most sensitive to equity issues in collaboration, and they tend to worry about whether the collaboration may be manipulated [70]. In addition, the emergence of collective action requires a common ideological basis [71]. An imbalance of power and resources will lead to different environmental goals, meaning that the actors will have differences in the degree and scope of environmental governance and the costs they are willing to pay. This can also lead to a non-linear increase in transaction costs relating to communication, decision-making, supervision, and other activities, and the effect of social incentives will gradually decline [72]. 

#### 2.2.3. Superior-Level Government Participation

A strong leader is needed to promote collaborative governance when the imbalance of power and resources is serious and participation incentive is insufficient [31]. Leaders can act as initiators of collaborative governance, introduce other actors into the collaborative network [56], and provide resource support or policy support for the operation of the collaborative network [32]. Leaders can promote the process of collaborative governance by formulating and maintaining rules, establishing organizations and trust relationships, facilitating dialogue, and exploring common interests [70]. Given the external constraints of China’s pressure-type centralized system design, local governments must execute the decisions of superior-level governments. From the perspective of the intrinsic motivation of the grassroots government, obedience to the superior-level government is more conducive to gaining advantages in the competition within the jurisdiction of the superior-level government. Therefore, the problems raised by the central government’s planning or policies are more obvious to the actors. The superior-level government also has a strong guidance and supervision function to the subordinate government. The laws, regulations, rules, and opinions formulated by the central government on the collaborative governance of the coastal zone environment can provide ideas and directions for grassroots environmental cooperation. Provincial or ministerial government can carry out close inquiry, instruction, regular review, supervision, vertical and horizontal communication, and coordination, playing the role of “assisting negotiation” in establishing collaborative relationships [61], thus allowing collaborative governance to become more authoritative, as well as reducing transaction costs and collaboration risks [73]. For example, due to the “10-year ban on fishing” in the Yangtze River, Shanghai City and Jiangsu, Zhejiang, and Anhui Provinces in the Yangtze River Delta have implemented joint supervision, coordinated legislation, and joint law enforcement. Led by Shanghai Municipal People’s Congress (NPC), the Standing Committee adopted a legal decision to promote and safeguard the ban on fishing. They jointly defined the areas and time limits for banning fishing, and regularly listened to work reports. In law enforcement, the three provinces and Shanghai built a joint platform for sharing ship registration information, active supervision of fishing vessels, retrospective supervision of aquatic product market circulation, and a platform for sharing law enforcement information. They carried out joint law enforcement against illegal and criminal acts that broke the ban on fishing. Overall, the ban on fishing led to good results. 

#### 2.2.4. Previous Cooperation Experience

The existence of trust capital is the starting point of voluntary cooperation [74], while trust comes from mutual understanding and identification [75]. It is a long and difficult process to establish trust between actors who are not familiar with each other. Past cooperation experiences can help actors to judge the credibility and legitimacy of partners, promote the establishment of trust relations and social capital, and bring about fuller communication and a higher level of commitment. In multiple interactions, actors will also establish cooperative attitudes and learn from each other’s social behaviors [76], which is conducive to shaping a concerted common vision, thus stimulating further cooperation in the future [77]. Successful cooperation experience means that there has been a relatively stable connection mechanism among the actors, the actors have mastered the key technologies required for collaborative governance, or the actors have formed a relatively mature policy system at a higher level, which will reduce the cost and difficulty associated with a new round of collaborative governance. Unsuccessful cooperation experiences may still contribute to the success of new partnerships [78]. Therefore, past cooperation experience implies the existence of trust and interdependence among actors. 

## 3. Research Design

### 3.1. Sample Selection

In recent years, the amount of land-based pollutants (mainly inorganic nitrogen, phosphate, oil hydrocarbons, organic matter, and heavy metals) carried into the sea in China’s coastal areas has reached more than 10 million tons per year [79]. About 15% of the rivers entering the sea in China are inferior to class V water quality, about 10% of the bay water is seriously eutrophicated, about 42% of coastal areas are overloaded with such resources, and more than 80% of the typical coastal ecosystem is in sub-healthy or unhealthy conditions. The coastal zone is where ecological environment problems are most concentrated and prominent in China. Among them, water areas inferior to class IV in the East China Sea are the most common in China. 

The coastal zone of the East China Sea starts from the North Bank of the Yangtze River Estuary in the north to the junction of Fujian and Guangdong provinces in the south, and passes through Shanghai, Jiangsu, Zhejiang, Fujian, and Taiwan. As data on Taiwan Province are not available at present, all of the 14 cities on the mainland coast of the East China Sea were chosen for this research. These cities are important windows into China’s rapid economic and social development. The development of agriculture, animal husbandry and fishery, reclamation of salt fields and planting, urban reclamation of land from the sea, port engineering and coastal construction, coastal heavy chemical industry agglomeration, aquaculture and coastal tourism, and so on, have led the coastal zone environment of the East China Sea to face serious problems, such as increased pollution and resource degradation. At the same time, although environmental supervision has always been a problem, considering factors such as multiple management, conflicting policies from different departments, and information barriers, the East China Sea coastal zone is still at the forefront of China’s reform and opening; therefore, the foundation for enterprises, social organizations, and the public to participate in governance is better. Therefore, we selected 14 typical cases of collaborative environmental governance in the 14 cities of the continental coastal zone of the East China Sea over the past five years, and analyzed the various configurations that promote the formation of medium–high intensity collaborative governance relationships.

The selection of cases was conducted according to the following five criteria. The first three criteria are theoretically-guided, while the fourth and fifth criteria are practically-guided.

(1) The selected cases have certain similarities, homogeneity, and comparability (i.e., there must be a similarity in background or characteristics between the cases, which are regarded as “constants” in the specific analysis). We required that all cases feature multiple actors participating in the environmental governance of coastal zone, with differing degrees of actor’s diversification in the sample overall. This may be viewed as a key case selection principle, as it would address the “many variables, small N”-problem;
(2) The selection of cases should have diversity at the same time, in order to ensure that the minimum number of cases is selected while achieving the greatest heterogeneity between cases. Cases in the sample overall have to differ significantly regarding the alleged causal conditions, e.g., both cases with limited costal environmental pollution and significant costal environmental pollution must be included in the overall sample, and both cases with limited previous cooperation experience and abundant previous cooperation experience must be included as well. This is needed in order to be able to detect multiple causal pathways to the outcome;
(3) The selected cases have both “maximum similarity” and “maximum difference,” where “maximum similarity” refers to grouping and pairing similar cases as much as possible in the process of the case study. The cases are selected according to the types of actors, e.g., the actors are only the government, state-owned enterprises or institutions; the actors include the government, private enterprises, social organizations, or individual citizens; the actors do not include the government, mainly social organizations, private enterprises, or individual citizens. Additionally, in each type, the similarity of collaborative governance actors is reflected, which can significantly improve the internal effectiveness of the observation relationship, while “maximum difference” refers to seeking the maximum heterogeneity in the selected cases. As the main actors of coastal zone environment collaborative governance are still the governments, they can be divided into central level, provincial and ministerial level, municipal level, and township level, so we select cases involving governments at different levels in order to ensure that the external effectiveness of the assumed causal relationship can be extended to the analysis.
(4) Only China’s cases were considered for inclusion in the sample. The decision to restrict the analysis to cases in China also reflects the fsQCA convention to select based upon a common context [80]. In addition, fsQCA relies significantly on the context knowledge of those conducting it [43]. The authors of this paper are most familiar with China’s context.
(5) Only cases for which significant amounts of information could be gathered were included. The analysis data in this paper include quantitative and qualitative data. Among them, the quantitative data include emissions data of major pollution sources in the coastal zone where each case is located. The data sources were China’s National Statistical Yearbook, the China Marine Ecological and Environmental Status Bulletin, local statistical yearbooks, and the public data from the Ministry of Ecology and Environment and local ecology and environment departments or bureaus. The qualitative data included the policy texts of the central, provincial, and municipal governments on coastal zone governance and the behavior data of multiple actors. The data sources were central and local government websites, news reports, and academic papers.

Basic information of the cases is provided in Table 2.

### 3.2. Method

Qualitative comparative analysis (QCA) is an analysis method based on set theory and Boolean Algebra [44,77,81,82]. It is suitable for small-scale samples with 4–7 variables and 10–40 samples [83]. The internal complexity of single cases can be combined with a systematic comparison of multiple cases. Combining the advantages of qualitative analysis and quantitative analysis, QCA involves the assumption that a certain state (outcome variable Y) is the result of the comprehensive effects of relevant influencing factors (multiple condition variables X), which can be used to compare and analyze the differences and commonalities of complex cases [84]. Through a certain number of cross-case comparisons, in the QCA method, Boolean algebra is applied to reduce the configuration of the combination of influencing factors, excavate various configurations that lead to the determination of the result variables, and distinguish between core conditions (i.e., necessary conditions) and non-core conditions (i.e., sufficient conditions) [81]. The combination of various conditions forms multiple necessary or sufficient configurations, constituting a causal path related to the outcome variables [55]. There are three common QCA methods: clear set qualitative comparative analysis (csQCA), multi-valued qualitative comparative analysis (mvQCA), and fuzzy-set qualitative comparative analysis (fsQCA). 

As there exists a subordinate relationship between the imbalance of power and resources in coastal zone environmental collaborative governance and the variable superior-level government participation, fsQCA can be considered better than mvQCA and csQCA in dealing with the degree and subordinate problems [85,86]. As such, in this paper, we utilize fsQCA. In fsQCA, every condition and result are a set, while every case has a membership score in each set. This process of assigning membership scores to cases is called calibration. To describe the actual situation, the result variable meeting the collaborative relationship does not conform to the two states of 0 and 1; instead, the variable values are determined as either 0, 0.33, 0.66, or 1 using the four-value scheme. The fsQCA 3.0 software (Ragin, Charles C. and Sean Davey. 2016. Fuzzy-Set/Qualitative Comparative Analysis 3.0. Irvine, California: Department of Sociology, University of California) is used for data analysis and processing [87]. 

### 3.3. Data Acquisition and Variable Measures

Coastal zone environment: The main indicators of inferior water quality in the coastal zone of the East China Sea are inorganic nitrogen and active phosphate. Pollution sources include land-based pollutants from rivers entering the sea, domestic sewage of coastal populations and industrial wastewater from industrial and mining enterprises, marine air pollutant deposition, marine garbage, and microplastics [88]. Therefore, the average values of the following indicators, before the establishment of the relationship between the actors in the cases, were selected to measure the environmental status of the coastal zone: ① the water quality category and chemical oxygen demand (COD), permanganate index, five-day biochemical oxygen demand, total phosphorus, dissolved oxygen, ammonia nitrogen, and other major excess indicators of monitoring section of the river entering the sea; ② the total discharge of sewage and major pollutants directly discharged into the sea; ③ the SO_2_ emissions; and ④ the total monitoring amount of floating garbage on the sea, garbage on the beach and seabed, and marine microplastic. SPSS cluster analysis was conducted to divide the pollutant emission values at case locations into four categories: low pollution, medium pollution, high pollution, and heavy pollution.

Balance level of power and resources: This was measured according to the difference in the level of actors. The level of an actor can be determined by the administrative level of government departments, institutions, and quasi-government organizations, and actors can be divided into central level, provincial and ministerial level, municipal level, and township level. Central enterprises and state-owned enterprises are classified according to their actual grading. Private enterprises, private non-enterprises, social organizations, civil associations, and individual citizens have no administrative level. Still, some social organizations have a government background, or the leaders of government departments concurrently serve as the leaders of social organizations. Thus, the level of a social organization refers to the government department that supports it. If the administrative levels of actors are arranged in a gradient from top to bottom, it is regarded as “quite unbalanced” of power and resources. When the administrative level of two or more actors is significantly higher than others, the situation is “basically unbalanced”; if only one actor has a significantly higher administrative level than others, the situation is “basically balanced”; and, if the administrative level of each actor is similar or the difference is not obvious, the situation is “quite balanced”. 

Superior-level government participation: Superior-level governments participate in joint meetings, leading groups, public forums, and collaborative groups of coastal zone environmental governance; supervise, inspect, and urge coastal zone environmental remediation; support governance by allocating special funds or formulating certain policies; and manage the behaviors of other actors participating in governance by ordering, commanding, controlling, and coordinating. The level of governments involved in governance can be divided as follows: non-superior government participation, urban government participation, provincial and ministerial government participation, and central and State Council government participation.

Previous cooperation experience: In China, economic development has been the top priority for a long time. Therefore, local governments, enterprises, social organizations, and citizens often start their cooperative relations from economic cooperation in the early stage. With the deepening of participatory governance in the social field, cooperation in social development affairs has increased. When the economy and society develop to a certain extent, environmental governance becomes a field that many actors pay close attention to. Therefore, the depth of previous cooperation experience can be measured from “shallow” to “deep,” according to whether the actors have played part in a framework or agreement for economic, social development, or environmental governance cooperation.

Now the fuzzy-set values to conditions for the different cases can be assigned. The assignment standard of variables was designed and assigned according to the four-value scheme (Table 3), usually adopted for the sub-dimensions at question with 0, 0.33, 0.66, and 1.00 to indicate “fully out,” “more out than in,” “more in than out,” and “fully in,” respectively [42]. 

We operationalize outcome variable “Y” via a review of press reports and scholarly literature. Drawing on the in-depth-explanation of the cooperation scale emphasized in the scholarly literature on collaborative governance [31], intergovernmental cooperation [89], public–private partnerships [22], and interlocal collaboration [24,54], the intensity of collaborative governance relationship can be described as three levels (Table 1), and “no relationship” should thus be assigned a value of 0. Then, the calibration is undertaken.

Conditional variable “X_1_” is measured by differences of coastal zone environment. We calculate the average value of the data of major excess indicators of monitoring section of the river entering the sea, the total discharge of sewage and major pollutants directly discharged into the sea, the SO_2_ emissions, the total monitoring amount of marine garbage and microplastic from China’s National Statistical Yearbook and China Marine Ecological and Environmental Status Bulletin; and then SPSS cluster analysis is used to divide the pollutant emission values of each case into four categories: low pollution, medium pollution, high pollution, and heavy pollution. Thus, these heavy pollution areas are coded 1 in the sample and served as a starting point for calibration.

Conditional variable “X_2_” is measured by differences in administrative levels of the actors. The anchor points are based on our knowledge of the theoretical concepts we aim to measure and the knowledge of the context of the cases. On one hand, in Chinese society, different administrative levels often mean different authority, responsibility, and financial resources available [90]. So, when the administrative level of main actors are similar, they will get similar power and resources, and the level of power and resources among them is balanced. Thus, these cases are coded 1. On the other hand, China’s administrative levels can be roughly divided into four levels: central level, provincial and ministerial level, municipal and county level, and township level. If the administrative levels of main actors are arranged in a gradient from top to bottom, it is obvious that the power and resources held by them are quite unbalanced, and thus, these cases are coded 0. If two or more actors have significantly higher administrative levels than other actors, it can be regarded as a basic unbalance of power and resources, and should be assigned a value of 0.33. If there is only one actor that has a higher administrative level than other actors, and other actors are at the same administrative level, it means a basic balance of power and resources, and thus should be assigned a value of 0.66.

Conditional variable “X_3_” is measured by the actor at the highest administrative level in the collaborative governance. Actors of a central level like deputy prime ministers or ministries and commissions that participate in collaboration, which indicates very strong participation, can be assigned a value of 1. So, according to the administrative sequence, if actors at the provincial and ministerial level like the Provincial Environmental Protection Department participate in collaboration, this indicates strong participation and they can be assigned a value of 0.66. If only municipal governments or functional departments participate, this indicates weak participation and they can be assigned a value of 0.33. If no superior levels of government participate in regional collaboration, the value is 0.

Conditional variable “X_4_” is measured by the type of cooperation that has been carried out. If a joint prevention mechanism or cooperation agreement on environmental affairs has previously been established, it can be assigned a value of 1 [61]. In China, the Central Committee put forward the guiding ideology of “taking economic construction as the center” in the early years of reform and opened up in 1980. Economic cooperation among multiple actors is more common, and local officials competing for economic growth are motivated by promotion incentives to make significant efforts to bid for investments and develop their economies. This may neglect secondary objectives such as environmental protection [91], so we assign the “cooperation history in economic affairs” as 0.33. With the deepening of reform and opening up, the social management function is becoming more and more important, and the collaborative governance experiences on social affairs has increased. As a kind of social public service, environmental governance has begun to be concerned by the collaborative governance actors [92], and we assign the “cooperation history in social affairs” as 0.66. Lastly, if there is no history of collaboration among all of the actors, the value is 0.

## 4. Results

### 4.1. Valued Scale of Variables

According to the variable assignment criteria of the four-value scheme shown in Table 3, combined with the actual data collected for the 14 cities, the values for each case were assigned as shown in Table 4. 

We used the fsQCA 3.0 software to analyze the assignment results. According to the variable assignment in Table 4, the combination configurations of influencing factors denoting weak, medium intensity, and high intensity relationships in coastal zone environmental collaborative governance were obtained.

### 4.2. Necessary Condition Inspection and Condition Configuration

#### 4.2.1. Analysis of Single Conditions

First, the consistency and coverage of the result variables under different conditions were calculated, in order to measure the causal relationships between the condition variables and the result variable. Consistency refers to the probability of the existence of condition variables when the result variables are reached; that is, the proportion of cases containing some condition variables in all cases reaching the results, indicating the consistency degree of cases displaying results under given conditions or a combination of conditions. Coverage refers to the probability of reaching the result variable in the presence of some condition variables and the degree of explanation of the result by conditions. Consistency measures the academic strength of the relationship between conditions and outcome variables, while coverage measures the empirical relevance of conditions.

Using the fsQCA3.0 software to calculate the consistency of variables (Table 5), the results indicated that the coverage values of condition variables including coastal zone environment, balance level of power and resources, superior-level government participation, and previous cooperation experience were all above 0.80, indicating a relatively reliable explanation of the result variable. The consistency of each single condition variable was lower than 0.9, indicating that there was no single sufficient and necessary condition for the generation of collaborative governance relationship of the coastal zone environment, from the perspective of complexity; instead, the result of the combined action of multiple variables must be considered. However, the consistency of each condition variable was greater than 0.60, indicating that the condition variables had a strong persuasive explanation effect on the result variable.

#### 4.2.2. Sufficiency Analysis

Based on constructing a multi-valued truth table with the variables, configuration analysis of the collaborative governance relationship was carried out in the fsQCA 3.0 software. When assessing conditional configuration using a truth table, it is necessary to set the case frequency and consistency threshold carefully. When using a small sample size, it is appropriate to set the frequency threshold of the case to 1.0; meanwhile, for the original consistency threshold, it is more appropriate to use a value of 0.8, according to the standard threshold of fsQCA 3.0. Through the Boolean minimization operation of the software, according to the inclusion of logical residual items, from non-inclusion to full inclusion, three kinds of results can be obtained: complex, reduced, and intermediate. As the intermediate solution has moderate complexity and does not allow for the elimination of necessary conditions, it is the most representative solution, with good explanation and universality [93]. Therefore, the intermediate solution was selected for configuration analysis. In addition, in the condition configuration sufficiency analysis, the core conditions of each solution were identified by comparing the nested relations between the intermediate solution and the reduced solution: the conditions that appear in both the intermediate solution and the reduced solution are the core conditions of the solution, while the conditions that appear only in the intermediate solution are the edge conditions. Therefore, the intermediate solution and the related configuration combination—that is, the combined configuration of influencing factors determining the strong relationship of collaborative governance for the coastal zone environment—were obtained, as shown in Table 6 and Table 7.

The outputs for the intermediate solution indicated that three combination configurations can lead to the establishment of medium–high intensity collaborative governance relationships among the multiple actors of coastal zone environmental governance. The coverage and consistency of the three paths were 0.932663 and 1.0, respectively, indicating that all the combinations of conditions could explain about 93.3% of the cases and had a high explanation degree. The consistency of all single paths (1–3) was 1.0, indicating that the combination of conditional variables in these three paths provided a good explanation of the result variables. Furthermore, the original coverage of the three paths was high, where the unique coverage of path 2 was slightly lower, indicating that paths 1 and 3 were more representative, to some extent.

### 4.3. Configuration Analysis of the Establishment of Collaborative Governance Relationship

#### 4.3.1. Path Dominated by Cooperation Experience and Balance of Power and Resources

The core conditions of Path 1 are the balance of power and resources and previous cooperation experience. The original coverage was 73.3% and the unique coverage was 26.7%. The most typical cases are the Qiantang River ecological coastal zone construction in Hangzhou, the Sandu’ao “Marine Pasture” environment comprehensive improvement action in Ningde, the beautiful bay construction of Xiamen southeast sea, the Dongshan Bay ecological restoration project in Zhangzhou, and the Minjiang Estuary wetland protection and restoration action in Fuzhou. These five cases involve different coastal environments. Nevertheless, the first four cases formed strong coastal environmental collaborative governance relationships (Y = 1), while Fuzhou also established medium intensity collaborative governance (Y = 0.66). Each case established a common goal of beautiful coastal zone construction or environmental remediation, jointly conducted construction or law enforcement actions several times, and established a regional coastal zone environmental governance coordination organization. For example, the Hangzhou municipal government specially established the “ecological coastal zone construction leading group and construction headquarters”; the Ningde Marine Fisheries Bureau, Public Security Bureau, Ecological Environment Bureau, Municipal Supervision Commission and Maritime Safety Bureau, and others jointly established the Ningde Municipal Joint Maritime Law Enforcement Group; the Xiamen Ecological Environment Bureau, Natural Resources Planning Bureau, Administrative Law Enforcement Bureau, Maritime Safety Bureau, Marine Police Station, Municipal Group, and others jointly set up the Municipal Marine Management Leading Group Office; and the Dongshan County Government, Procuratorate, Court, Ecological Environment Bureau, Natural Resources Planning Bureau, Marine Fisheries Bureau, and others set up a special group for Dongshan Coral Nature Reserve. These joint working groups presented clear organizational empowerment, promoted all departments to sign the administrative agreement on collaborative governance, and supervised the implementation of the agreement to achieve the normalization of collaborative governance and law enforcement. These high intensity relationships of collaborative governance were mainly due to the balance of power and resources among various actors, as well as past cooperation experience in many environmental governance affairs.

The actors of coastal zone environmental governance in Hangzhou and Ningde were mainly urban government departments with equal power and state-owned enterprises, and marine fishery institutions with resource strength equivalent to government departments; meanwhile, Xiamen, Zhangzhou, and Fuzhou presented more diverse actors in bay environmental governance. For example, in addition to the municipal Ecological Environment Bureau, the Natural Resources Planning Bureau, the Administrative Law Enforcement Bureau, and other government departments, Xiamen also has the third National Oceanographic Institute, Fujian Oceanography Institute, Fujian Key Laboratory for Coastal Ecology and Environment Studies, Xiamen Key Laboratory of Water Resources Utilization and Protection, Xiamen University, and other scientific research institutions; public institutions such as the Water Conservancy Project Quality and Safety Station, Urban Planning and Design Institute, national nature reserves, and national marine parks; and social organizations such as the Blue Ribbon Marine Protection Association. Although the actors differ, the administrative levels of government departments, scientific research institutions, and public institutions remain the same. Scientific research and public institutions also occupy the academic and technical resources of scientific governance in marine environmental governance. Although the Blue Ribbon Marine Protection Association does not have an administrative level, it is the largest marine public welfare organization in China with the largest number of volunteers, in which enterprises and the public are the main members. There is no “commander” among the multiple actors who occupies a strong position and coerces the weak side, such that the actors are more open to each other; this is also more conducive to the multi-directional information interaction of governments, enterprises, universities, research institutions, and users.

At the same time, the multiple actors in these cases also had cooperation experience in environmental governance, forming a spontaneous horizontal coordination ability. For example, the water control offices of Qiantang District, Xiaoshan District, and Binjiang District involved in the Qiantang River section of Hangzhou Bay have previously set up a normalized three-district joint meeting and a joint law enforcement inspection group, signed a three-district joint protection and governance agreement, and held joint meetings once a year and at any time when major problems were encountered. This joint meeting invited relevant departments of the municipal government and construction enterprises to focus on the co-governance of the Qiantang River section of Hangzhou Bay, informed and shared the joint governance and law enforcement of the Qiantang River, and studied the promotion of key projects. In 2018, Ningde carried out the “thousand-person action” of the “sea cleaning campaign”, comprising 17 marine-related local district government departments as well as public security, armed police, border defense, maritime, port administration, and administrative law enforcement units, in order to carry out 24-h patrol supervision and cleaning of illegal aquaculture, floating garbage, and abandoned fish rafts in the Sandu’ao sea area. As early as 1994, Xiamen established a highly coordinated “leading group for integrated coastal zone management,” led by the mayor, marine-related department officials, and marine experts, in order to cooperate in relation to aquatic products, shipping, environmental protection, ports, and other matters. Scientific research institutions and government-affiliated institutions also give full play to the supporting role of marine science and technology research. In cooperation with government departments, they have implemented actions such as the “bridge instead of the embankment to invigorate water,” “beach restoration and reconstruction project,” and “replanting mangroves.” The leaders and volunteers of the Blue Ribbon Marine Protection Association also overlap with the above departments involved in the actions. Long-term cooperation has established a good trust relationship and social capital among actors, formed the common vision of jointly building a beautiful coastal zone, and provided the actors with advanced cooperation skills through their cooperation experiences. In this way, a certain mechanism of risk- and benefit-sharing has been formed.

#### 4.3.2. Path Dominated by the Coastal Zone Environment and Balance of Power and Resource

The core conditions of Path 2 are the coastal zone environment and the balance level of power and resources. The original coverage was 36.7%, while the unique coverage was 6.6%. As such, the representativeness of this path was slightly weaker. Typical cases are the Hangzhou Bay marine ecological environment protection in Shaoxing and the “one island, one station” clean beach action in Zhoushan. 

These two cases are located in areas with some of the most serious environmental pollution in the East China Sea. According to the China Marine Ecological and Environmental Status Bulletin from 2016 to 2020, the East China Sea has the widest area with lower than class IV water, comprising more than twice the total areas with inferior to class IV water in the Bohai Sea, the Yellow Sea, and the South China Sea. These lower than class IV waters are mainly distributed in the Yangtze River Estuary, Hangzhou Bay, and Zhejiang coast areas, and the main substances exceeding the associated standards are inorganic nitrogen and active phosphate, resulting in severe eutrophication. Unlike the strong participation of superior leaders in the cases of Ningbo, Taizhou, Jiaxing, and Wenzhou—which are also high-pollution areas—the actors of marine ecological environment protection in Shaoxing of Hangzhou Bay are mainly the Shaoxing Ecological Environment Bureau, Water Conservancy Bureau, and other government departments directly responsible for marine environmental management, as well as the communities and industrial parks, printing and dyeing plants, chemical plants, sewage treatment plants, livestock and poultry farms, and ecological pastures directly related to the marine environmental pollutants in Shangyu District, Yuecheng District, and Zhuji City along the coast. The actors in the clean beach action in Zhoushan presented non-government characteristics, being mainly composed of social organizations, volunteers, sea-related units, and individuals, while the Zhoushan Ecological Environment Bureau provided necessary assistance. The level of power and resources among the actors in each case was relatively balanced, and a medium intensity collaborative governance relationship (Y = 0.66) was established, giving play to the advantages of multiple actors while realizing the integration of government resources and the linkage of governance behaviors.

Specifically, under the guidance of the municipal government departments, the industrial park management office, and park enterprises, Shaoxing implemented the transformation and upgrading plan for the printing, dyeing, chemical, and electroplating industries. Five printing and dyeing clusters have been built in Shangyu District, accumulating chemical enterprises from Yuecheng District. At the same time, the reconstruction of sewage pipe networks and the construction of centralized sewage treatment facilities in the industrial parks and living communities was carried out. The sewage treatment plant also installed flowmeters and online monitoring facilities connected with the municipal Ecological Environment Bureau, in order to regularly publicize basic information and ensure compliance of sewage outlets into the sea. Livestock and poultry breeding plants participated in offline grid patrol and online collaborative prevention and control, improved their sewage and fecal storage facilities, and pastures have been implemented with farmland nitrogen and phosphorus ecological interception ditches. All actors have established a clear common pollution control goal of reducing the discharge of pollutants (e.g., inorganic nitrogen and active phosphate) to Hangzhou Bay, and have accordingly implemented joint remediation actions.

Zhoushan has a small land area and a large sea area. This city has 1390 large and small islands, accounting for 20% of China’s islands. It is an important location for the marine fishery industry. There are six national marine ranch demonstration areas, accounting for 55% of the number of national marine ranch demonstration areas in Zhejiang Province. The extensive construction and use of artificial reefs and fishing boats in fishing ports have led to a large amount of marine fishery waste discharge pollution into the coastal zone [94,95]. These pollutants damage the marine ecological environment, restrict the production income of local fishers, and affect human health through the food chain. The Zhoushan Thousand Islands Marine Environmental Protection Public Welfare Development Center, Marine Environmental Protection Association of Zhejiang Ocean University, environmental protection volunteers, owners of the coastal aquaculture industry, fishing ports, fishing boat owners, and other related units and individuals have participated in the remediation action. The wives of fishers joined together to urge the fishers to take fishery garbage back to shore for recycling; volunteers and sea users jointly cleaned up beach garbage on the Zhoushan Islands and jointly participated in a hearing on marine fishery waste disposal. The enterprises and individuals improved their power and resource possession levels by investing in environmental treatment funds or joining social organizations. It can be seen that extremely serious coastal environmental pollution will bring tension to the local people, especially when it endangers the economic and social benefits of local sea users, even without the intervention of superior-level leaders at the provincial, ministerial, or central level. Without deep cooperation experience, a medium intensity collaborative governance relationship can still be established among the local governments and other stakeholders of the marine environment, presenting a balanced level of power and resources.

#### 4.3.3. Path Dominated by the Coastal Zone Environment, Cooperation Experience, and Balance of Power and Resources

The core condition of Path 3 is the coastal zone environment, while the marginal conditions are superior-level government participation and previous cooperation experience. The original coverage was 49.8%, and the unique coverage was 13.4%. The representativeness of this path was at the medium level. Typical examples include Jiaxing illegal farming cleanup and coastal remediation, Taizhou Sanmen Bay marine environment remediation action, Wenzhou Yueqing Bay “one bay, one policy” remediation action, and Ningbo Xiangshan Port “Blue Bay” renovation project. Among them, cases in Jiaxing, Taizhou, and Wenzhou formed medium intensity collaborative governance relationships (Y = 0.66), while the case in Ningbo formed a high intensity collaborative governance relationship (Y = 1).

These four places are also located in areas with the serious environmental pollution in the East China Sea. The central government also presented a great degree of intervention. For example, the cases in Taizhou, Wenzhou, and Ningbo involved the Central Ecological and Environmental Protection Inspection Group, which is the highest institution of environmental protection governance supervision in China. It usually supervises and inspects the implementation process of local government environmental protection policies through field investigation, individual conversations, acceptance of petition letters, data review, open and secret visits, and so on. Before supervision, the inspection group visited seawalls and grasslands in order to investigate the ecological protection of water, sludge storage yards, sea sluice gates, underground rivers, and the ecological protection of coastal waters. They traced flows to investigate the pollution sources, conducted secret investigations, collected sufficient clues on relevant environmental pollution problems, and fixed the evidence chain. This served to force the local government to actively respond to the environmental problems found by the inspection group, and systematically integrated multiple administrative departments into the “inspection” or “rectification” team under the unified management of the local party and government leaders, forming an active community and fully integrating the scattered administrative resources. At the same time, the Ministry of Finance and the Ministry of Natural Resources also jointly allocated funds for marine ecological protection and restoration to support coastal remediation and restoration, supporting coastal wetland vegetation planting and restoration, bay water pollution control, coastal structure cleaning and dredging, ecological corridor construction, and so on. The subsidy funds reached the level of 100 million yuan. An adequate supply of funds can fill the gap between enterprises, social organizations, and government departments. In the case of Jiaxing, the National Marine Environment Monitoring Center is the pre-eminent institution deeply involved in the environmental governance of coastal zones. This center is a public institution directly under the Ministry of Ecology and Environment. The center specially set up a resident assistance working group in Jiaxing. The working group discussed the technical problems, key directions, and key tasks in the process of coastline remediation with various departments of local government, and jointly discussed and formulated the implementation plan for coastline repair.

In addition, the actors in these four cases generally had extensive experience in social or environmental governance cooperation. Jiaxing, Taizhou, Wenzhou, and Ningbo have fully implemented the three-level “beach (bay) head” system at the municipal, town, and village levels since 2017, and the responsibility for the environmental governance of the coastal zone is contracted by regions and assigned to the “beach (bay) head”. The beach (bay) heads at the town and village levels are assumed by the secretaries of the Party Communities of the town and village, and the beach (bay) head positions at the municipal level are held by the leader of the municipal Party Committee or municipal government, or the leader of the Ecological Environment Bureau, the Natural Resources and Planning Bureau, and other administrative departments related to the marine environment. The “beach (bay) head” system has formed a cooperative relationship that considers the linkage between the river and the sea at both the upper and lower levels. It has also attracted the intellectual support of Ningbo University, Ningbo Institute of Oceanography, and other universities and scientific research institutions, laying a good foundation for establishing multi-agent synergy in the following actions of coastal zone environmental remediation.

## 5. Discussion

**Figure 2:**
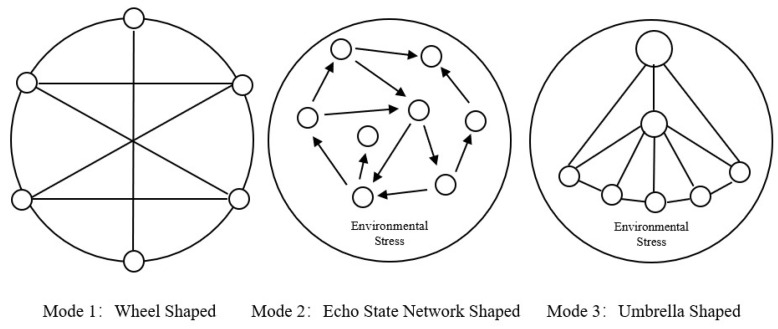
Three modes facilitating medium–high intensity collaborative governance relationships. Note: “○” represents the actors participating in the coastal zone environmental governance; “─” represents the establishment of a medium–high intensity collaborative governance relationship among actors; and “→” represents the initialization stage of the establishment of medium–high intensity collaborative governance relationship, where the direction of the arrow represents the direction of the randomly generated connection relationship.

From the perspective of single influencing factors, there was no necessary condition. We found that when the power and resources among actors are relatively balanced, when a relatively harsh coastal environment is faced, or when actors have previous cooperation experience, the participation of superior-level government is unnecessary. In these cases, local non-governmental actors can organize themselves to establish medium–high intensity collaborative governance relationships. When the power and resources among actors are unbalanced and the coastal environment is relatively harsh, the superior-level government must intervene vigorously to promote the establishment of medium–high intensity collaborative relationships and resolve the ecological environment problems as soon as possible. Accordingly, we can utilize three modes to successfully establish medium–high intensity collaborative governance relationships (Figure 2).

### 5.1. Wheel-Shaped Mode

In this mode, the power and resources among actors are relatively balanced and form a complementary relationship with previous cooperation experience, promoting the formation of a high intensity collaborative governance relationship. The balance of power and resources eliminates the hierarchical contradictions of multiple actors and command constraints in the collaborative process. The previous experience of cooperation in environmental affairs eliminates the uncertainty in new environmental governance affairs. These two elements form a “wheel” structure in the practice of governance, which promotes in-depth communication among actors; improves information and technology sharing through joint meetings, leading groups, and other forms; defines the mechanism of collaboration in specific environmental governance activities; establishes a special coordination organization to manage collaborative governance affairs; and promotes the continuous operation of the collaborative “wheel.”

### 5.2. Echo State Network-Shaped Mode

In this mode, local economic and social development is more sensitive to the marine environment. When facing an extremely high level of environmental pollution, even if there is a lack of experience regarding cooperation in environmental governance or social governance in the past, actors may also form a medium–high intensity collaborative governance relationship, if their power and resources are relatively balanced. Like an echo state network, the pressure of ecological destruction and environmental pollution in the coastal zone is the “input unit,” creating a high-dimensional complex dynamic state-space for the governance actors. In this context, the actors with balanced power and resources are the neurons in the reserve pool. At the very beginning, the neurons are not connected, but connections are randomly generated in the initialization stage of the network, which are strengthened in the training of the whole echo state network, thus forming a consensus on the collaborative governance rules. The practice of systematic governance, involving aspects such as joint law enforcement, beach cleaning, and other specific joint actions, is the final output.

### 5.3. Umbrella-Shaped Mode

In this mode, local governments also face serious environmental pollution in coastal areas, but the power and resources among actors are unbalanced. There are two or more actors whose administrative levels are significantly higher than other actors, or the levels of power and resource of actors are graded from top to bottom. However, a medium–high intensity collaborative governance relationship is still constructed. The reason for this is that the superior-level government plays a strong role in management, guidance, cohesion, and promotion. Especially when the Central Ecological and Environmental Protection Inspection Group is involved, the top leaders of a province or a city can strongly intervene in the governance behavior of local departments in the form of welcoming evaluation and promoting construction, thus playing a core communication role. Leaders and inspectors at the central level can also establish contacts with grassroots actors, in order to investigate and directly point out the sources of environmental damage in coastal areas. Meanwhile, as these actors have also had close exchanges and cooperation in the past, they can quickly establish trust in the face of environmental issues raised by superior-level leaders, effectively implementing environmental improvement actions in coastal areas.

The results of this analysis correspond in large part to expectations on the basis of theory and research in other countries, but add new insights highlighting the importance of collaborative governance among multiple stakeholders in coastal zone environments.

First, it was confirmed that, in the aspect of collaborative governance relationship formation regarding coastal zone environmental or water pollution control, there are some similar conditional variables between the East China Sea and other coasts in the world. For example, in Central Europe (Rhine, Meuse, Scheldt, Ems, Elbe, Po) and on the Iberian Peninsula (Ardour-Garonne/Cantabrico Oriental, Douro, Guadiana), high superior-level government participation promotes a high degree of integration in the realm of water resources management, and high previous cooperation experience has promoted the management of water quality in the Torne River, shared between Sweden, Finland, and Norway, as these states have a history of deep economic integration and considerable collaboration in the water realm [77]. Path 3 proposed in our paper (high pollution of coastal zone environment × high superior-level government participation × high previous cooperation experience) is consistent with this result.

Second, some case studies conducted in marine protected areas of Australia, Indonesia, Vietnam, Italy, France, the U.K., Spain, Brazil, and so on have shown that, rather than the “command-and-control” approach inherent in top-down hierarchies with high superior-level government, their roles have been adapted to provide governance direction through persuasion, partnerships, markets, communities, and associations [12,13]. In this line, economic, communication, knowledge, legal, and participation incentives can be used to promote decentralization in collaborative governance. Path 1 proposed in this paper (high balance level of power and resources × high previous cooperation experience) is consistent with these cases.

Third, environmental challenges, such as the coastal areas being contaminated with solid waste, sewage, industrial effluents, chemical run-off from agriculture, and wastes from the transportation sectors in the Caribbean Sea, are naturally connected with environmental issues. Therefore, a polycentric governance approach has been adopted in the Caribbean, which integrates various actors with a special focus on empowering local communities at the island level. A key factor in effective governance relies on a system that is island- and community-based, but also connected and coordinated at the regional level, where their government is only one of the players [16]. Path 2 proposed in our paper (high pollution of coastal zone environment × high balance level of power and resources × low superior-level government participation) is consistent with this study.

In summary, this paper details the combination configurations of different conditional variables that promote the formation of collaborative relationships, which complements the deficiencies associated with research considering only single influencing factors. Although this paper focused on the coastal zone of the East China Sea, it is closely linked with a global perspective, and provides three paths that can be selected, according to local conditions, for the collaborative governance of coastal zones in other regions.

## 6. Conclusions

In this paper, we aimed to study the combinations of factors that can facilitate the establishment of medium–high intensity coastal zone environment collaborative governance relationships. The collaborative governance relationship strength was divided into four levels: “no relationship,” “sharing information, establishing the goal of collaboration,” “forming a collaborative mechanism, carrying out joint actions,” and “forming special collaborative organizations and normative agreements.” Based on the classical research theory of collaborative governance, we propose four key influencing factors: “coastal zone environment,” “balance level of power and resources,” “superior-level government participation,” and “previous cooperation experience.” The fsQCA approach was applied to study typical cases of environmental governance in 14 cities on the mainland coast of the East China Sea, and we determined the combination configurations affecting the establishment of collaborative governance relationships. In particular, we obtained three combination paths, the results of which showed a high degree of overall consistency and coverage. Combined with relevant cases, the theoretical mechanisms of different paths were fully demonstrated. The results also indicate that the fuzzy-set qualitative comparative analysis method is effective for analyzing the factors influencing the establishment of coastal zone environment collaborative governance relationships. The key contributions of this paper are as follows.

First, we complemented the current scholarly consensus that “Environment conditions,” “Balance level of power and resources,” and “Previous cooperation experience” are necessary conditions regarding the emergence of collaborative governance relationships. However, unlike the existing quantitative studies, which have mostly considered the single influencing factor of collaborative governance, we utilized fsQCA to analyze the combinations of conditional variables affecting the formation of collaborative governance relationship. We sorted out typical cases of environmental governance in the coastal zone of the East China Sea, in order to examine the starting conditions for establishing collaborative governance relationships among middle- and high-level governments, local governments, social organizations, sea-related enterprises, and individuals under China’s unique administrative authoritarian system. The empirical research determined three combined paths leading to the formation of medium–high intensity collaborative governance, and summarized three cooperation modes.

Second, our findings challenge the universality conclusion that the leadership of superior-level of government is an absolutely necessary condition for playing a core role in collaborative governance. We found that the involvement of superior-level leaders is not always necessary for multiple actors to conduct collaborative governance. Even under such an administrative system as that in China, when the coastal environmental problems faced by local actors are very prominent, if the actors present a relatively balanced level of power and resources, they may still form a medium intensity collaborative governance relationship, even with no previous cooperation experience. Furthermore, when the environmental pollution in the coastal zone is at a medium or low level, actors with relatively balanced power and resources who have prior experience in social governance or environmental governance cooperation can also form medium–high intensity collaborative governance relationships.

Third, we believe these findings are not only of scholarly interest, but potentially also of great practical relevance. We collected new environmental data and environmental governance actors’ information of 14 cities along the continental coastal zone of the East China Sea. Scholars are struggling with the difficulty in obtaining high-quality data for examining collaborative governance relationships. The inspiration from small-N studies and complex causal configurations allows us to examine the influence of environmental data and actors’ information on the formation of collaborative governance relationship. This may be an important step of mitigating the dilemma of establishing cooperative relationships in order to avoid looming serious environmental governance problems in many coastal cities.

In general, this paper provided empirical evidence regarding the starting conditions for collaborative governance of coastal zone environments, including different modes by which multiple actors participate in governance, and established a collaborative mechanism to deal with coastal zone environmental problems. We also provided three paths as reference for promoting the participation of social organizations, enterprises, citizens, and so on.

From a methodological perspective, the fsQCA method employed in this paper appears to be a valuable addition to the methodological toolbox for international coastal environment governance research. It allows for qualitatively mapping of the complex causal relationships between conditions, which may be difficult to access by quantitative inquiry. The consideration of cases in 14 cities in the coastal zone of East China Sea further supported a more nuanced, context-sensitive account of the outcome.

This study still had some research limitations: First, most of the data were second-hand data obtained through open networks. Although the integrity of the information has been confirmed repeatedly, it still may be subject to selective bias. In-depth interviews and fieldwork can supplement this deficiency in further research. Second, in terms of research methods, fsQCA can provide researchers with a set of methods to explore the relationships between conditional variables and outcome variables, but it does not provide a complete causal explanation (particularly in terms of the calibration of conditions and the interpretation of results), and there may exist other explanatory variables, which can be supplemented through the use of a variety of research methods in the future. Third, the specific work of collaborative environmental governance in the coastal zone of the East China Sea is still in its infancy. The collaborative governance mode at this stage cannot represent the collaborative governance mode in all regions over the world in the future. However, it still has certain reference significance at the current stage. In the future, we will continue to track and study existing cases, as well as expand the scope of the research objects.

## Figures and Tables

**Table 1:** Measurement of the intensity of coastal zone environment collaborative governance relationships.

Collaborative Relationship Intensity	Measurement Criteria	Reference
Weak collaborative relationship	Information sharing and exchange exist among actors; actors have established abstract collaborative goals, but there is no specific collaborative action.	Li, Koppenjan and Verweij, 2016 [55] Keast and Mandell, 2014 [52]
Medium intensity collaborative relationship	Actors have formed a preliminary consensus on the rules and mechanisms of collaborative governance. Joint actions have emerged, such as joint enforcement, resource exchange, and so on.	Mandell and Keast, 2008 [51] Koontz and Steelman, 2004 [53]
High intensity collaborative relationship	Actors have changed the traditional governance mode of working independently and set up special transactional or coordinating agencies to manage collaborative governance issues; standardized collaborative governance protocols have been formulated.	Bryson, Crosby, and Stone, 2015 [56] Terman, Feiock, and Youm, 2019 [54]

**Table 2:** Basic information of the cases studied to assess the construction of environmental collaborative governance relationship in the East China Sea coastal zone.

No.	City	Case	Actors
1	Shanghai	Preservation and restoration of reed damage in Dongtan Wetland, Nanhui (2019–2022)	Central Ecological and Environmental Protection Inspection Group, National Forestry and Grassland Administration, Shanghai Greening and City Appearance Administration (Shanghai Forestry Administration), Shanghai Lingang Special Area Management Committee, China Biodiversity Conservation, and Green Development Foundation, experts and scholars from Shanghai Ocean University, bird-watching enthusiasts
2	Jiaxing	Illegal farming clean-up and coastal remediation (2018–2020)	National Marine Environment Monitoring Center, Jiaxing Ecological Environment Bureau, Natural Resources and Planning Bureau, Agriculture and Rural Affairs Bureau, Water Resources Bureau, Aquatic Technology Promotion Station, Marine Ecological Breeding Base, “coast chief” at City, Town, and Village
3	Hangzhou	Qiantang River ecological coastal zone construction (2021–2022)	Hangzhou Municipal Government Ecological Coastal Zone Construction Leading Group and Construction Headquarters, Water Control Office of Qiantang District, Xiaoshan District and Binjiang District (Three District Joint Conference, Joint Law Enforcement Inspection Group), state-owned development companies, large central enterprises
4	Shaoxing	Hangzhou Bay marine ecological environment protection (2021–2022)	Shaoxing Ecological Environment Bureau, Water Resources Bureau, urban residential areas and industrial parks of Shangyu District, Yuecheng District, and Zhuji city, printing and dyeing enterprises, chemical enterprises, sewage treatment plants, livestock, and poultry farms, ecological pastures
5	Ningbo	Xiangshan Port “Blue Bay” renovation project (2018–2020)	Central Ecological and Environmental Protection Inspection Group, Ministry of Finance, Ministry of Natural Resources, State Oceanic Administration, Zhejiang Marine Fisheries Bureau, Ningbo Marine Fisheries Bureau, Ningbo Ecological Environment Bureau, Xiangshan County Government, Xiangshan Marine Fisheries Bureau, Yinzhou District government, Yinzhou Marine Fisheries Bureau, Jiushan Islands National Nature Reserve, Hua’ao Island National Marine Park, Ningbo Institute of Oceanography, Ningbo University, coastal villages and communities
6	Zhoushan	“One island, one station” clean beach action (2017–2022)	Zhoushan Thousand Islands Marine Environmental Protection Public Welfare Development Center, Zhoushan Ecological Environment Bureau, Zhejiang Ocean University Marine Environmental Protection Association, aquaculture owners, fishing boats, fishing ports, environmental protection volunteers, fishermen
7	Taizhou	Sanmen Bay marine environment remediation action (2021–2022)	Central Ecological and Environmental Protection Inspection Group, Ministry of Finance, Ministry of Natural Resources, Zhejiang Provincial Government, Taizhou Municipal Government, Sanmen County Party Committee, Sanmen Government and Inspection Office, Sanmen Natural Resources and Planning Bureau, Ecological Environment Bureau, Agriculture and Rural Affairs Bureau, Housing and Urban-Rural Development Bureau, Administrative Law Enforcement Bureau, Transportation Bureau, Economic and Information Bureau, Environmental Commission, Sanmen Bay Maritime Department, Park Management Committee, coastal villages and communities
8	Wenzhou	Yueqing Bay “One bay, one policy” remediation action (2021–2022)	Central Ecological and Environmental Protection Inspection Group, Ministry of Finance, Ministry of Natural Resources, Yueqing Ecological Environment Bureau, Natural Resources Planning Bureau, Agriculture and Rural Affairs Bureau, Administrative Law Enforcement Bureau, Housing and Urban-Rural Development Bureau, Municipal Utility Construction Center, Transportation Bureau, Ecological Environment Bureau, Yueqing Bay Maritime Department, coastal villages and communities
9	Ningde	Sandu’ao “Marine Pasture” environment comprehensive improvement action (2020–2021)	Ningde Marine Fisheries Bureau, Public Security Bureau, Ecological Environment Bureau, Supervision Commission, Maritime Safety Bureau, Leisure Marine Fishery Demonstration Department, Marine Sanitation Institutions
10	Fuzhou	Minjiang Estuary wetland protection and restoration action (2017–2021)	The Fuzhou Commissioner’s Office of the National Forestry and Grassland Administration, Fujian Wetland Protection Center, Wildlife and Wetland Resources Monitoring Center, Wetland Research Center, Fuzhou Forestry Bureau, Fuzhou Association for Science and Technology, Minjiang Estuary Wetland Nature Reserve Management Office, villagers (full-time wetland keeper), Mangrove Foundation, HeYi Institute, Fujian Normal University, Key Laboratory of the Coastal and Wetland Ecosystems (Xiamen University), Wetland Protection Obligation Publicity Cycling Team
11	Putian	Meizhou Bay “Protect blue bay, build beautiful North Bank” beach clearing action (2019–2021)	Management Committee of Meizhou Island in Putian, Mazu Public Welfare Organization, merchants along the harbor of Meizhou Island, volunteers
12	Quanzhou	Procuratorial public interest litigation action of “Protecting Quanzhou coastline” (2020–2021)	Quanzhou Procuratorate, Law Enforcement Team of Quanzhou Marine Fisheries Bureau, marine wetland ecological grid member of street communities
13	Xiamen	Beautiful bay construction of Xiamen southeast sea (2020–2021)	Xiamen Ecological Environment Bureau, Natural Resources Planning Bureau, Administrative Law Enforcement Bureau, Maritime Safety Bureau, Marine Police Station, Municipal Group, Water Conservancy Project Quality and Safety Station, Urban Planning and Design Institute, Marine Environmental Sanitation Management Station, Rare Marine Species Nature Reserve, Xiamen National Marine Park, the third National Oceanographic Institute, Fujian Oceanography Institute, Fujian Key Laboratory for Coastal Ecology and Environment Studies, Xiamen Key Laboratory of Water Resources Utilization and Protection, Xiamen University, Blue Ribbon Marine Protection Association
14	Zhangzhou	Dongshan Bay ecological restoration project (2021–2022)	Zhangzhou Ecological Environment Bureau, Dongshan County Government, Dongshan Procuratorate, Court, Ecological Environment Bureau, Natural Resources Planning Bureau, Marine Fisheries Bureau, Guangzhou Waterway Bureau Co., Ltd., Zhangzhou Blue Carbon Judicial Protection and Ecological Governance Research Center, marine environment experts (ecological environment technical investigator), marine environmental protection public organizations, fishing ports, and village communities

**Table 3:** Variables and calibration regarding economic cooperation, social development cooperation, and environmental governance cooperation.

Variables	Calibration & Description			
	0	0.33	0.66	1.00
Y: Intensity of collaborative governance relationship	No relationship	Sharing information, establishing the goal of the collaboration	Forming a collaborative mechanism, carrying out joint actions	Forming special collaborative organizations and normative agreements
X_1_: Coastal zone environment	Low pollution	Medium pollution	High pollution	Heavy pollution
X_2_: Balance level of power and resources	Quite unbalanced	Basic unbalanced	Basic balanced	Quite balanced
X_3_: Superior-level government participation	No superior government participation	Municipal government participation	Provincial and ministerial government participation	Central and State Council government participation
X_4_: Previous cooperation experience	No cooperation history	Cooperation history in economic affairs	Cooperation history in social development affairs	Cooperation history in environmental governance affairs

**Table 4:** Values for the conditions and outcome of the considered cases.

No.	City	Y	X_1_	X_2_	X_3_	X_4_
1	Shanghai	0.33	0.33	0	1	1
2	Jiaxing	0.66	1	0.66	0.66	0.66
3	Hangzhou	1	1	1	0.33	1
4	Shaoxing	0.66	0.66	1	0.33	0.66
5	Ningbo	1	1	0	1	1
6	Zhoushan	0.66	0.66	0.66	0.33	0
7	Taizhou	0.66	1	0.66	1	0.66
8	Wenzhou	0.66	0.66	0.66	1	0.66
9	Ningde	1	0.66	1	0.33	1
10	Fuzhou	0.66	0	0.66	0.66	1
11	Putian	0.33	0	1	0	0.33
12	Quanzhou	0.33	0	0.66	0.33	0
13	Xiamen	1	0.33	1	0.33	1
14	Zhangzhou	1	0.33	1	0.33	0.66

**Table 5:** Consistency and coverage table of conditional variable.

Variables	Consistency	Coverage
X1	0.698492	0.910878
X2	0.866332	0.865462
X3	0.631156	0.823067
X4	0.866332	0.895119

**Table 6:** Intermediate solution for the outcome.

Model: Y = f(X_1_, X_2_, X_3_, X_4_) Algorithm: Quine–McCluskey configuration			
--- INTERMEDIATE SOLUTION ---			
Frequency cutoff: 1 Consistency cutoff: 1 Assumptions:			
Configuration	Raw coverage	Unique coverage	Consistency
X_2_ × X_4_	0.732663	0.267337	1
X_1_ × X_2_ × ~X_3_	0.366834	0.066332	1
X_1_ × X_3_ × X_4_	0.498493	0.133668	1
solution coverage: 0.932663 solution consistency: 1			

**Table 7:** Combination configuration of influencing factors for the medium–high intensity collaborative governance relationship.

Variables	Path 1	Path 2	Path 3
X_1_		●	●
X_2_	●	●	
X_3_		⊗	·
X_4_	●		·
Consistency	1	1	1
Raw coverage	0.732663	0.366834	0.498493
Unique coverage	0.267337	0.066332	0.133668
Solution consistency	1		
Solution coverage	0.932663		

Note: Big black circles (●) indicate that a core condition exists, small dots (·) indicate that an edge condition exists, and small circles with a cross (⊗) suggest a lack of edge condition. A blank space indicates that this condition is irrelevant in the results.

## Data Availability

The data presented in this study are available on reasonable request from the corresponding author.
